# Data Rights and Responsibilities: A Human Rights Perspective on Data Sharing

**DOI:** 10.1177/1556264615591558

**Published:** 2015-07

**Authors:** Theresa L. Harris, Jessica M. Wyndham

**Affiliations:** 1 American Association for the Advancement of Science, Washington, DC, USA

**Keywords:** human rights, data, ethics, right to science, privacy, informed consent, Article 15

## Abstract

A human-rights-based analysis can be a useful tool for the scientific community and policy makers as they develop codes of conduct, harmonized standards, and national policies for data sharing. The human rights framework provides a shared set of values and norms across borders, defines rights and responsibilities of various actors involved in data sharing, addresses the potential harms as well as the benefits of data sharing, and offers a framework for balancing competing values. The right to enjoy the benefits of scientific progress and its applications offers a particularly helpful lens through which to view data as both a tool of scientific inquiry to which access is vital and as a product of science from which everyone should benefit.

The enormous potential benefits of sharing data, only now becoming possible as a result of new technologies, are driving demands for researchers to make their data openly accessible. As the articles in this special issue point out, while researchers are committed to achieving the scientific advances that can result from data sharing and reuse, data sharing raises important ethical questions regarding privacy, confidentiality, and informed consent. Human rights advocates around the world share these same concerns.

Risk-based ethics reviews inform essential protections for research subjects, including those involved in studies where the resulting data are made openly available. Privacy, confidentiality, and informed consent, central to a risk-based assessment, are also aspects of a human-rights-based analysis. However, several unique characteristics of human rights have prompted some researchers to look at this framework of shared rights and responsibilities as an alternative to a risk-based analysis. For example, Knoppers, Harris, Budin-Ljøsne, and Dove (2014) have recommended developing an international code of conduct based on human rights to guide genomic and clinical data sharing. Similarly, Duke and Porter (2013) have found the international human rights framework helpful for understanding the responsibilities involved in data sharing. In both articles, the authors found that human rights offer a cross-border perspective, a socio-legal context, and a holistic approach that helps answer some of the broader ethical questions that the sharing of massive, and more easily analyzed, databases raises.

- **Human rights are a shared, internationally recognized framework**. The Universal Declaration of Human Rights (UDHR), adopted unanimously by the United Nations in 1948, arose from World War II as a global statement of the dignity of all people and the limitations of governments. Many of the principles articulated in the UDHR and later codified in the core international human rights treaties are directly applicable to the conduct of research: individual autonomy, bodily integrity, limits on government power over the decisions of individuals, the right to information, and informed consent as an essential means of protecting individual rights (United Nations Educational, Science and Cultural Organization [UNESCO], 2005). Around the world, these shared global human rights standards have been implemented in national constitutions and domestic legal structures, including regulatory requirements for research. As such, these are not just moral statements of principle; in many parts of the world, these are legally binding obligations on researchers. The complex webs of legislation, regulation, and professional codes applicable to data sharing, thus, have common underpinnings based in international human rights principles (Vizzini, 2015). As such, human rights can provide a foundation for collaborative research that crosses jurisdictions and inform the development of much-needed harmonized standards for scientific collaboration (Leshner & Turekian, 2009).
- **Human rights call for a legal and political system that protects researchers and research subjects.** A fundamental purpose of all international human rights treaties is to articulate obligations of governments to each other and to their respective citizens. Thus, analyzing the human rights implications of data sharing, both positive and negative, reaches beyond individual ethical responsibilities and includes an evaluation of government responsibilities. Consequently, a human rights based approach considers everyone involved in the data life cycle and considers their respective roles and responsibilities in the process. A human rights based approach to responsible data sharing must recognize the obligations of the government to have legal structures in place to protect those rights and hold those who violate those rights accountable for their wrongful actions. This includes access to justice, access to information, due process for claims of injury, accountability for those who unjustly commit harm (including, and especially, government officials), and reparations for victims.
- **Human rights put limits on government power and also establish expectations for positive actions.** Human rights standards are, first and foremost, government obligations to protect individuals from mistreatment and discrimination. For example, the UDHR and several human rights treaties require governments to prohibit experimentation on persons without informed consent. But human rights treaties also oblige governments to respect and fulfill certain rights by providing access to shared resources and ensuring that every individual can meet their basic needs and live in dignity. Among these obligations are the right to enjoy the benefits of scientific progress and its applications, a right that is intertwined with many other rights and that has special relevance to the question of data sharing (Chapman & Wyndham, 2013). The potential relevance of “the right to science” to the ethical questions around data sharing is discussed in more detail below.
- **Human rights laws recognize individual *and* community rights.** International human rights law recognizes that, alongside individual rights, communities can hold rights too. Community rights are often associated with the rights of indigenous peoples, but other types of communities also have rights to their cultures and to shared resources such as communal lands, environmental resources, and medical knowledge. In many ways, enforcing community rights is very similar to enforcing individual rights; informed consent, due process, and accountability are just as essential for protecting community rights as for individual rights. However, the means for deciding what constitutes a community’s informed consent or determining who can bring forward a claim on behalf of a community must be considered.
  The concept of community rights is particularly relevant to concerns about the potential impacts of aggregated biometric and genomic data. On the positive side, framing the question of “who benefits” in terms of community rights may help clarify some of the murkier issues in population-based research. At the same time, even when an individual’s data can be anonymized, indigenous groups or other ethnic minorities may be at risk of stigmatization, discrimination, exploitation, or other harms that may only occur when the data from members of the community are aggregated. Community rights also have implications for informed consent processes, which usually focus on the individual, where in some social structures, community-wide consent reaching beyond the sampled research subjects may be needed (Tsosie & McGregor, 2007).
- **Human rights laws address the tensions between interrelated values.** As implied by the previous paragraphs, there are tensions between individual rights and community rights, and other rights, while interrelated, have inherent tensions as well. For example, the right to personal privacy can sometimes conflict with the right to enjoy the highest achievable standard of health (Privacy International, 2013). The UDHR was designed to provide an interdependent system of rights that balance each other while recognizing the sometimes competing interests between individual rights and societal goals, protecting minorities within systems where majorities make shared decisions. Our understanding of how to weigh these tensions is constantly evolving as new questions are considered by international human rights bodies and analyzed by legal scholars. As a result, the body of human rights law and scholarship offers the scientific community a wealth of resources to guide decision making about how to make difficult choices between seemingly competing rights and responsibilities (Scheinin, 2013).

## Data Sharing Through a Human Rights Lens

At this point, many of the potential harms that could arise from data sharing are still speculative. They anticipate technology that does not yet exist. For example, re-identification due to aggregation is generally not possible with the technology currently available. However, most agree that it will be possible—and there are cases that already indicate that day is coming soon (Gymrek, McGuire, Golan, Halperin, & Erlich, 2013). Scientists engaged in efforts to develop shared standards for responsible data sharing to prevent future harms may find that applying a human rights approach reframes some of the central questions (Knoppers et al., 2014). A human rights approach helps highlight a different set of problems, priorities, and solutions.

- What rights do individuals have to their own data? In what ways could those rights be violated?
- What responsibilities do governments have to protect individual rights to data? How do these translate to private actors, including researchers?
- How are community rights to data defined? Who can give informed consent to those rights? Are those rights potentially in conflict with individual data rights?
- Are there situations—for example, public emergencies such as epidemics—in which the right to one’s own data can be abrogated?
- How are data rights connected to other rights: freedom from discrimination, the right to association, the right to family, and the right to due process?
- What due process procedures need to be in place for a just system of data sharing? What are the potential harms? What would be an appropriate remedy for those harms? Who should be held accountable for injuries?
- What rights do individuals and communities have to access the research findings that result from their data? What are their rights to benefit from the findings?
- What processes and procedures need to be in place to protect those rights? For example, could those who eventually suffer from genetic discrimination as a result of a disclosure bring a claim in court or through an administrative process? Depending on the country, even if some members of society do have such access, a member of a marginalized group may not (Privacy International, 2013). In that case, the genetic discrimination reinforces an existing human rights abuse. Researchers may need to ask similar questions about the ability to provide proper informed consent, whether due to individual vulnerability or group marginalization (Human Rights Watch, 2011).

## The Right to “Enjoy the Benefits of Scientific Progress and Its Applications”

One right in the overarching human rights framework that may be instructive when considering data sharing is the right to “enjoy the benefits of scientific progress and its applications” as recognized in Article 15 of the “International Covenant on Economic, Social and Cultural Rights” (United Nations, 1966). Not directly concerned with data and not the only right of relevance, this right does nonetheless offer one lens through which to view data as both a tool of scientific inquiry to which access is vital and as a product of science from which everyone should benefit.

As a tool of scientific inquiry, the traditional and primary community that benefits from data is the scientific community. Indeed, in the context of a discussion about the meaning of Article 15, a multi-disciplinary group of scientists recognized that access to data is important for “(1) on-going research potential; (2) reproduction of results for validation; (3) longitudinal comparisons; (4) training and education of the next generation of scientists; and (5) historic value” (AAAS Science & Human Rights Coalition, 2013, p. 8). As such, data are integral to the scientific process and to scientific progress.

To view data sharing from this perspective is to start from the premise that data generation, storage, and dissemination are necessary for scientific progress to continue and should be facilitated by government as part of their human rights obligations. However, when viewed primarily as a tool of scientific inquiry, is it arguable that access to data is not a universal right to be enjoyed equally by everyone? Certainly, access to data can be legitimately circumscribed based on risk and, many scientists argue, scientific socialization (AAAS Science & Human Rights Coalition, 2013). What are the implications of this interpretation for the public’s access to data generally and to their own data specifically? Article 15 does not provide the answers and requires consideration of the broader human rights framework.

What the language of Article 15 does clearly indicate is that the findings derived from rigorous scientific data analysis should be applied for the benefit of everyone. From climate change to the effectiveness of vaccinations, everyone has the right to benefit from the data that address these and other issues of significance to society. To achieve this goal, as well as continued scientific progress, Article 15 suggests that the processes and regulatory systems established to manage data collection, storage, and dissemination should be open and transparent for, without that, access to data is meaningless.

## Conclusion

Applying human rights to data sharing will not answer all the ethical questions that open sharing and reuse raise. In fact, it raises some new questions for consideration. What a human rights based analysis can offer is a different lens through which to view those questions, one that connects the policies and practices regarding data sharing to the people involved throughout the data cycle, as well as the socio-political context in which it takes place. The human rights framework provides a shared set of values and norms that cross borders, cultures, and local legal systems; defines rights and responsibilities of the different actors involved in contributing, analyzing, and sharing data; addresses both the harms and the benefits of data sharing; and outlines principles for balancing competing interests. A human rights analysis can thus serve as a useful tool for the scientific community and policy makers as they develop harmonized international norms and complementary national policies for data standards and research protocols.
